# Sea Urchin Food Waste into Bioactives: Collagen and Polyhydroxynaphtoquinones from *P. lividus* and *S. granularis*

**DOI:** 10.3390/md22040163

**Published:** 2024-04-03

**Authors:** Margherita Roncoroni, Giordana Martinelli, Stefano Farris, Stefania Marzorati, Michela Sugni

**Affiliations:** 1 Department of Environmental Science and Policy, University of Milan, Via Celoria 2, 20133 Milan, Italy; margherita.roncoroni@unimi.it (M.R.); giordana.martinelli@unimi.it (G.M.); michela.sugni@unimi.it (M.S.); 2 Department of Food, Environmental and Nutritional Sciences, University of Milan, Via Celoria 2, 20133 Milan, Italy; stefano.farris@unimi.it

**Keywords:** sea urchins, collagen, polyhydroxynaphthoquinones, biomaterials, wound healing, circular economy

## Abstract

Approximately 75,000 tons of different sea urchin species are globally harvested for their edible gonads. Applying a circular economy approach, we have recently demonstrated that non-edible parts of the Mediterranean Sea urchin *Paracentrotus lividus* can be fully valorized into high-value products: antioxidant pigments (polyhydroxynaphthoquinones—PHNQs) and fibrillar collagen can be extracted to produce innovative biomaterials for biomedical applications. Can waste from other edible sea urchin species (e.g., *Sphaerechinus granularis*) be similarly valorised? A comparative study on PHNQs and collagen extraction was conducted. PHNQ extraction yields were compared, pigments were quantified and identified, and antioxidant activities were assessed (by ABTS assay) and correlated to specific PHNQ presence (i.e., spinochrome E). Similarly, collagen extraction yields were evaluated, and the resulting collagen-based biomaterials were compared in terms of their ultrastructure, degradation kinetics, and resistance to compression. Results showed a partially similar PHNQ profile in both species, with significantly higher yield in *P. lividus*, while *S. granularis* exhibited better antioxidant activity. *P. lividus* samples showed higher collagen extraction yield, but *S. granularis* scaffolds showed higher stability. In conclusion, waste from different species can be successfully valorised through PHNQ and collagen extraction, offering diverse applications in the biomedical field, according to specific technical requirements.

## 1. Introduction

About 75,000 tons of sea urchins are consumed annually worldwide [1,2], and the main consumer countries include Japan, Chile, New Zealand, the Philippines, and Europe—especially Mediterranean countries, such as Italy, France, and Spain [3]. Since sea urchins are marketed exclusively for their edible gonads, the rest—i.e., test, spines, and viscera (70–90% of the animal mass)—becomes restaurant and food industry waste. In a circular perspective, these parts can still be exploited to obtain precious molecules and high added value products. It was previously shown that this waste (mainly test and spines) can be a source of polyhydroxynaphthoquinones (PHNQs; [4,5,6,7,8,9,10,11,12,13,14]). These are sea urchins’ specific secondary metabolites belonging to the naphthoquinone class [14]. Given their biological properties (e.g., antioxidant activity, antimicrobial activity), the extraction of these molecules for potential medical applications has received increasing attention [4,10,12,15,16,17]. For example, the antiallergic effects of PHNQs were reported in animal models such as guinea pig ileum contraction, rabbit eye allergic conjunctivitis, and rabbit local skin irritation [18]. Furthermore, glycopeptide fraction (GPF) from internal organs of green sea urchins showed potent anti-inflammatory effects, especially for the treatment of bronchitis [19]. More specifically, the PHNQ echinochrome A was introduced as an active substance of the drug Histochrome, commonly used in ophtalmological and cardiological clinical practice in Russia [20]. In the test and spines of sea urchins, there are six most common and abundant PHNQs: echinochrome A (EchA), spinochrome A (SpA), spinochrome B (SpB), spinochrome C (SpC), spinochrome D (SpD), and spinochrome E (SpE) [6,9,10,11,12,13,14]. However, some sea urchin species may contain additional pigments, such as specific structural analogues [6]. Besides monomeric PHNQs, spinochrome dimers (e.g., anhydroethylidene-6,6′-bis(2,3,7-trihydroxynaphthazarin), its isomer and ethylidene-6,6′-bis(2,3,7-trihydroxynaphthazarin) were identified [21]. 

Recently, it has also been shown that tissues from sea urchin waste are a source of high-value native collagen that was used to develop innovative collagen-based biomaterials for tissue regeneration [22,23,24,25]. Marine collagens are known for their low antigenicity and high biodegradability [26,27], useful in various applications. A particularly promising area is tissue regeneration, with the production of three-dimensional scaffolds [28,29]. Although numerous studies have been searching for alternative marine collagen sources [30,31,32]—such as fish [33,34,35], sponges [36], and jellyfish [33,37]—collagen extracted from the echinoderms sea urchins has been studied and tested for years as a highly promising biomaterial [22,23,24] because it can be easily extracted without destructive methods, preserving its native fibrillar conformation, including its glycosaminoglycan surface decoration, structural integrity, and mechanical performance [22]. Until now, sea urchin collagen has only been extracted and biotechnologically exploited from the edible Mediterranean–Atlantic species *Paracentrotus lividus* (Lamarck, 1816), specifically from the peristomial membrane, an echinoderm exclusive and unique dynamic collagenous tissue (i.e., mutable collagen tissue, MCT) surrounding the mouth [38,39,40]. 

Since both collagen and PHNQs from sea urchin waste are potentially useful in the biomedical, pharmaceutical, and cosmetic fields, their extraction and utilization represent an important starting point for a more sustainable future in waste management and a circular economy approach.

Approximately 40 edible species of sea urchins are exploited worldwide. In particular, the species of *Strongylocentrotus* sp., i.e., *Strongylocentrotus droebachiensis* (Müller, 1776), *Strongylocentrotus franciscanus* (A. Agassiz,1863), and *Strongylocentrotus intermedius* (A. Agassiz, 1864) are the species with the highest global demand [41]. They are harvested primarily by Russia, Japan, the United States, and Canada. Other common edible species are *Pseudocentrotus depressus* (A. Agassiz, 1864) in Korea and Japan, *Heliocidaris crassispina* (A. Agassiz, 1864) in Australia, *Evechinus chloroticus* (Valenciennes, 1846) in New Zealand, *Tripneustes gratilla* (Linnaeus, 1758) in the Indo-Pacific, *Psammechinus miliaris* (Müller, 1776) in the Northeast Atlantic, *Paracentrotus lividus,* and *Sphaerechinus granularis* (Lamarck, 1816) in the Atlantic and Mediterranean regions [3]. 

Given the wide variety of edible sea urchins that are marketed annually, the aim of this work was to investigate whether different edible species other than *P. lividus*, which make up the global sea urchin market and therefore global waste, could be exploited to obtain antioxidants and collagen, and whether these molecules could be efficiently used for eco-friendly biotechnological purposes. Therefore, we selected another common Mediterranean Sea urchin species, *S. granularis*, and conducted a comparative study with *P. lividus* in terms of PHNQ and collagen extraction as well as the production of collagen-derived biomaterials. 

While *P. lividus* is the most commonly harvested mediterranean sea urchin [42] and it is found at depths of up to 40 metres, *S. granularis* is found at depths up to 130 m [43]. *P. lividus* typically does not exceed 7 cm in test diameter [44], and although it is commonly known as the purple sea urchin, the colour of its long and thin spines can vary from black to reddish-brown, dark brown, light brown, purple, or green. *S. granularis,* on the other hand, can reach a test diameter of up to 13 cm [45] and is characterised by short and thick spines, which can be completely white or purple with occasional white tips [46].

In this work, PHNQs were extracted from the restaurant wastes of both sea urchin species, *P. lividus* and *S. granularis*; PHNQs were identified, and their relative abundances were compared using Ultra Performance Liquid Chromatography—Photodiode Array coupled to Mass Spectrometry (UPLC-PDA-ESI-HR-MS). In addition, the antioxidant activities were evaluated. For the *S. granularis* species, antioxidants were extracted separately from tests with purple or white spines to identify any intraspecific differences between sea urchins with different pigmentations. 

Collagen was extracted from both species, its yield was determined, and the collagen was used to produce scaffolds. The ultrastructural features of the biomaterials were then compared using scanning electron microscopy (SEM). Porosity, degradation kinetics, swelling ratio, and mechanical resistance to compressive stress were also evaluated.

## 2. Results

### 2.1. PHNQs 

#### 2.1.1. PHNQ Extraction

**Figure 1:**
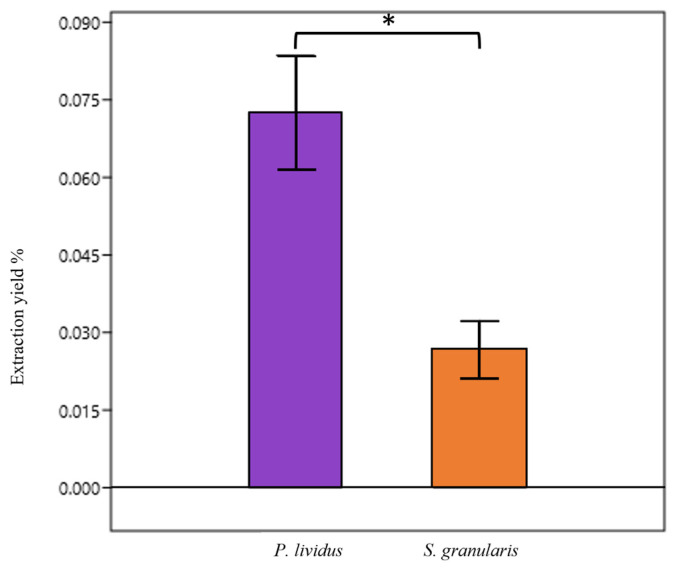
Yield of PHNQ extraction (% means ± st. dev) from the waste of the two species, *P. lividus* and *S. granularis* (both purple and white). * *p* < 0.05, Mann–Whitney test.

The PHNQ mean extraction yield from *P. lividus* was significantly higher (Mann–Whitney, *p* < 0.05) than that of *S. granularis*: 0.07 ± 0.02% (mean ± st. dev) *vs* 0.026 ± 0.015% (Figure 1). More specifically, the mean PHNQs yield of purple *S. granularis* was 0.033 ± 0.02%, while the mean PHNQs yield of white *S. granularis* was lower, 0.015 ± 0.01%.

#### 2.1.2. PHNQ Identification and Quantification

**Figure 2:**
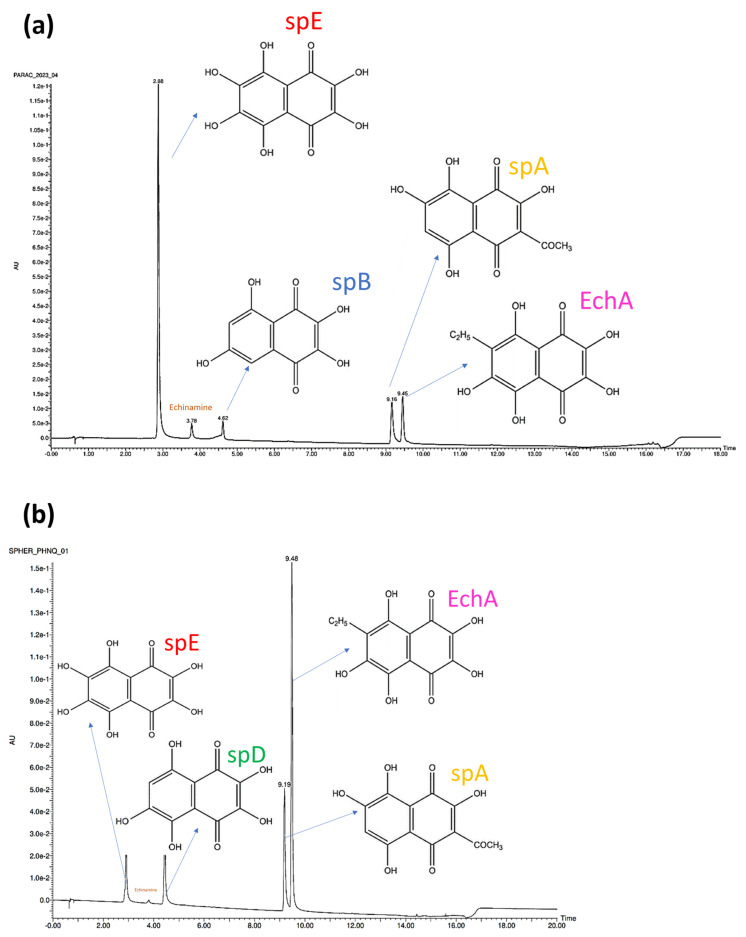
UPLC-PDA chromatograms of *P. lividus* (**a**), *S. granularis* (**b**) at 445 nm detection wavelength.

The PHNQs found in *P. lividus* were identified by UPLC-PDA-ESI-HR-MS as follows (Figure 2, t_R_ = retention time): spinochrome E (SpE, t_R_ = 2.9 min, *m*/*z* = 252.9988), spinochrome B (SpB, t_R_ = 4.6 min, *m*/*z* = 221.0085), spinochrome A (SpA, t_R_ = 9.2 min, *m*/*z* = 263.0190), and echinochrome A (EchA, t_R_ = 9.5 min, *m*/*z* = 265.0350). Among the less common PHNQs, echinamine (t_R_ = 3.78, *m*/*z* = 252.0145) was also identified. 

The PHNQs found in *S. granularis* (both purple and white) were identified as follows (Figure 2): spinochrome E (t_R_ = 2.9 min, *m*/*z* = 252.9986), spinochrome D (t_R_ = 4.4 min, *m*/*z* = 237.0032), spinochrome A (t_R_ = 9.2 min, *m*/*z* = 263.0192), and echinochrome A (t_R_ = 9.5 min, *m*/*z* = 265.0346). Echinamine was also identified exclusively in the purple variety. The ESI-HRMS spectrum of each peak is provided in the Supplementary Materials (Figures S1–S6).

SpE, SpA, and EchA were found in both sea urchin species, while SpB and SpD were species-specific. The relative ratios of PHNQs from *P. lividus* in each analysed extract vary depending on the batch. In Table 1 PHNQ relative percentages were calculated on the basis of area under each peak in the 445 nm chromatogram. Three injections were performed for each sample and the areas variations differed by less than 1%. EchA co-elutes with spA, so they were considered together. The results for *S. granularis* are more consistent (generally EchA/SpA > SpE > SpD), but all extractions were made from a single batch.

#### 2.1.3. ABTS^∙+^ Assay

**Figure 3:**
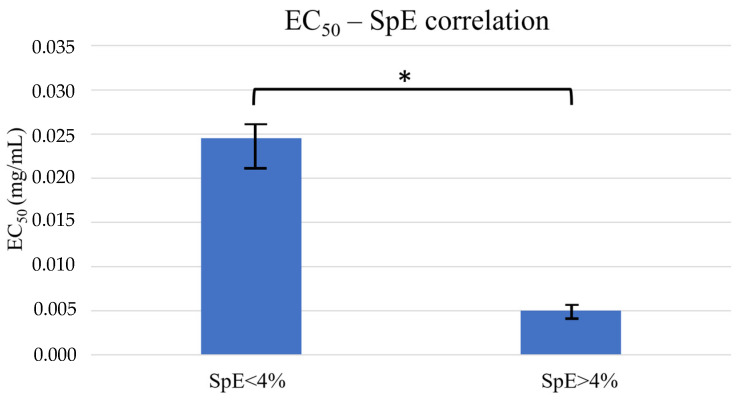
EC_50_ (means ± st. dev) correlated with % SpE. It is shown that PHNQ mixtures containing high percentages of SpE (>4%) were significantly (* *p* < 0.05, Mann–Whitney) characterised by lower EC_50_ values compared to mixtures with low percentages of SpE (<4%) regardless of the sea urchin species.

The antioxidant activity of the PHNQs extracted from both species was determined as EC_50_ and then correlated with the relative ratios of the PHNQs. The EC_50_ values were on average 0.02 ± 0.01 mg/mL for *P. lividus* and 0.005 ± 0.002 mg/mL for *S. granularis* regardless of the colour of the spines (Table 1). These values were comparable to that of Trolox^®^ (0.0035 ± 0.0005 mg/mL), an analogue of vitamin E commonly used in the literature as a reference antioxidant, indicating that the mixtures obtained have rewarding antioxidant capacity. PHNQ extracts containing high percentages of SpE (>4%) displayed significantly lower (Mann–Whitney, *p* < 0.05) EC_50_ values, ten times lower than those of mixtures with low SpE percentages (<4%), regardless of the sea urchin species (Figure 3). As high percentages of SpE were more frequently found in *S. granularis* extracts, this species generally showed higher antioxidant activity.

### 2.2. Collagen

#### 2.2.1. Collagen Extraction

**Figure 4:**
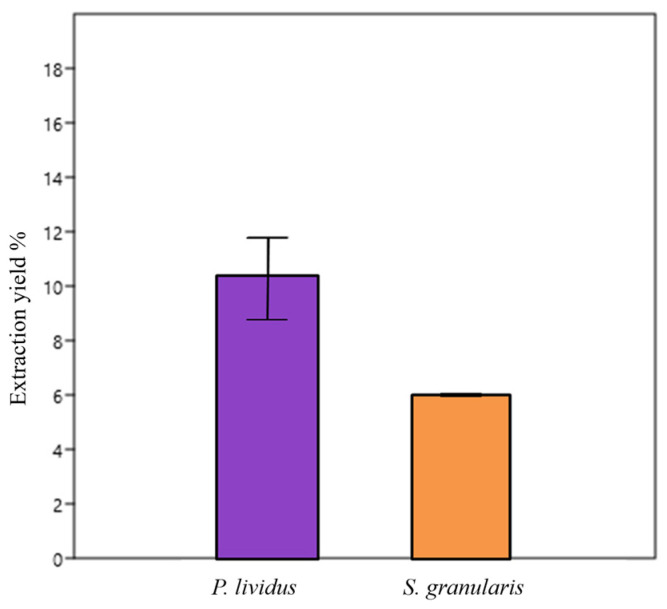
Collagen extraction yield (% means ± st. dev) from the waste material of the two species, *P. lividus* (10 ± 2.5%) and *S. granularis* (6%).

The mean collagen extraction yield of *P. lividus* was 10 ± 2.5% (mean ± st. dev), while the collagen extraction yield of *S. granularis* was lower: 6% (non-significant χ2 test), (Figure 4). The amount of collagen extracted was 17 mg from *S. granularis* and 7.75 ± 1.3 mg from *P. lividus*.

#### 2.2.2. Ultrastructural Analysis

**Figure 5:**
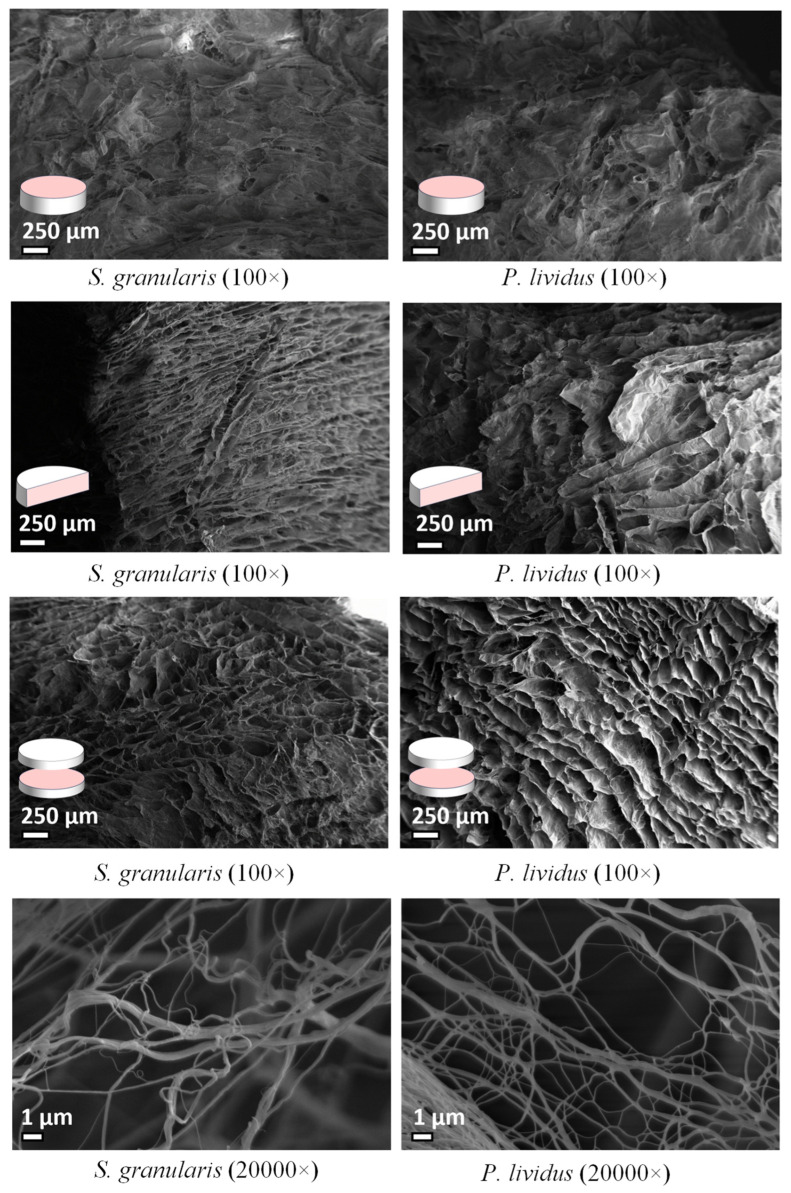
SEM images of *S. granularis* and *P. lividus* scaffolds displaying the spongy structure and a network of fibrils organised to form interconnected pores visible in the upper surface, cross section, and longitudinal section. The last two SEM pictures show fibrils magnification.

Overall, there was no evident difference in the structural organization between the scaffolds produced from collagen of the two sea urchin species. In both cases, the scaffold appeared as a spongy structure and a network of fibrils organised to form interconnected pores. However, analyses of the cross section of *S. granularis* scaffolds revealed a more regular arrangement of horizontal pores and an apparently more compact structure. The diameter of the fibrils range was 64–442 nm for *P. lividus* and 109–441 nm for *S. granularis*, with mean values significantly lower (Mann–Whitney, *p* < 0.05) in *P. lividus* (201.12 ± 79 nm) compared to *S. granularis* (249.31 ± 80 nm) (Figure 5).

#### 2.2.3. Macroporosity

**Figure 6:**
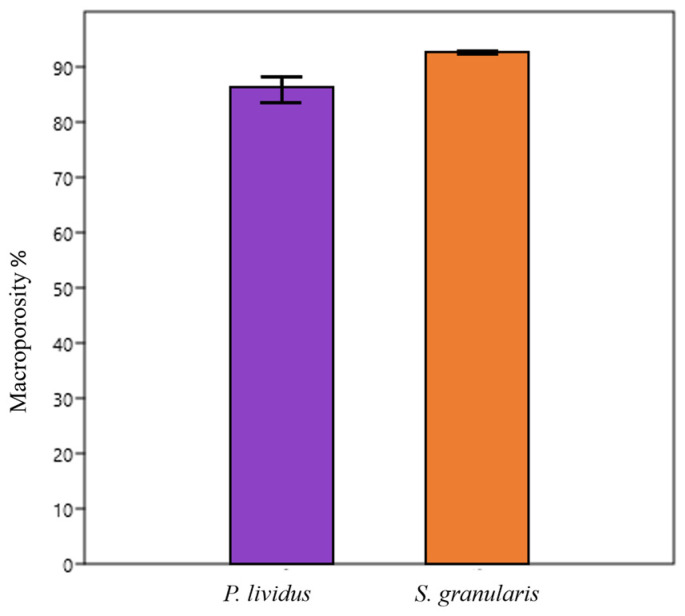
Scaffold microporosity values (%) (means ± st.dev) of *S. granularis* and *P. lividus*, 92.47 ± 0.6% and 85.59 ± 4.5%, respectively. The difference was not statistically significant (Mann–Whitney, *p* > 0.05).

Although the scaffolds of *S. granularis* had a slightly higher average percentage of macroporosity compared to those of *P. lividus* (92.47 ± 0.6% and 85.59 ± 4.5% respectively), the difference was not statistically significant (Mann–Whitney, *p* > 0.05), (Figure 6).

#### 2.2.4. Degradation Tests

**Figure 7:**
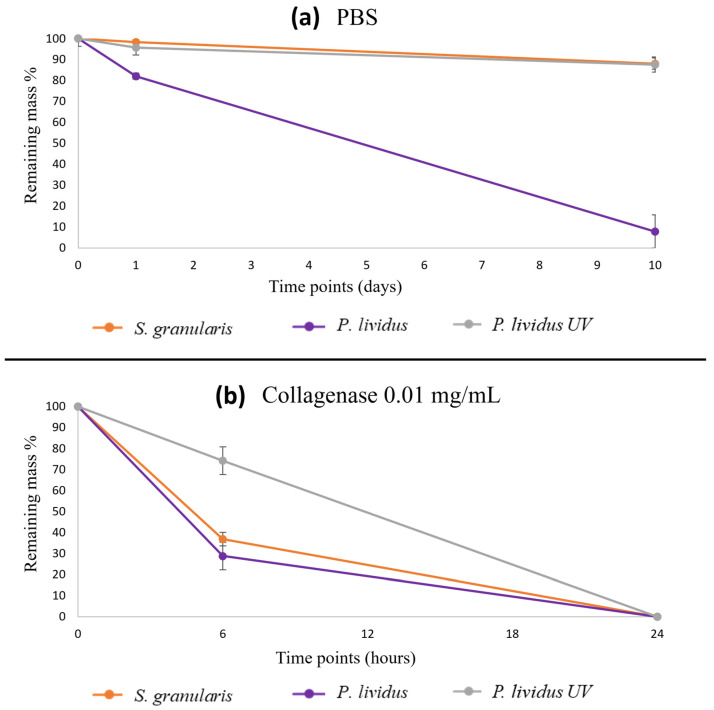
(**a**) Degradation kinetics of the *S. granularis* and *P. lividus* scaffolds, the latter also UV-crosslinked (average remaining mass % ± st.dev): *S. granularis* scaffolds were more stable, and they showed a similar degradation kinetics to those of *P. lividus* crosslinked scaffolds. (**b**) Degradation kinetics of the *S. granularis* and *P. lividus* scaffolds, the latter also as UV-crosslinked scaffold (average remaining mass % ± st. dev). Significance values are shown in the text. The statistics are reported in Tables S4 and S5 in the Supplementary Materials.

After 1 day in PBS, the average remaining masses were 98.5% and 72.4% for *S. granularis* and *P. lividus,* respectively (Kruskal–Wallis, *p* > 0.05). After 10 days, the average remaining masses of the scaffolds were 88% and 8% for *S. granularis* and *P. lividus,* respectively (Kruskal–Wallis, *p* < 0.05). Thus, *S. granularis* scaffolds were more stable, and they showed similar degradation kinetics to those of scaffolds crosslinked with *P. lividus*: after 10 days, both samples showed an average remaining mass of 88% (Figure 7).

In collagenase treatment, after 6 hours, no significant difference was observed (Kruskal–Wallis, *p* > 0.05) between *P. lividus* and *S. granularis* scaffolds, with average remaining masses of 29% and 37%, respectively. However, both samples degraded significantly more (Kruskal–Wallis, *p* < 0.05) compared to cross-linked *P. lividus* scaffolds (Figure 7). After 24 hours, all the samples were completely degraded. 

#### 2.2.5. Hydrations Tests/Swelling Properties

**Figure 8:**
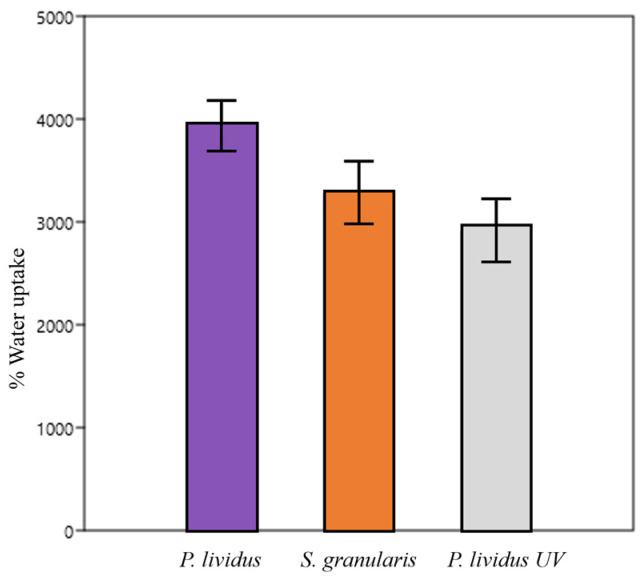
Water uptake (%) ± st. dev for *P. lividus*, *S. granularis*, and UV-cross-linked *P. lividus* scaffolds.

Water uptake was not different (Kruskal–Wallis, *p* > 0.05) between *P. lividus*, *S. granularis*, and cross-linked *P. lividus* scaffolds. The absorbed solution percentages were, on average, 3951 ± 510%, 2902 ± 715%, and 3271 ± 711%, respectively (Figure 8). 

**Figure 9:**
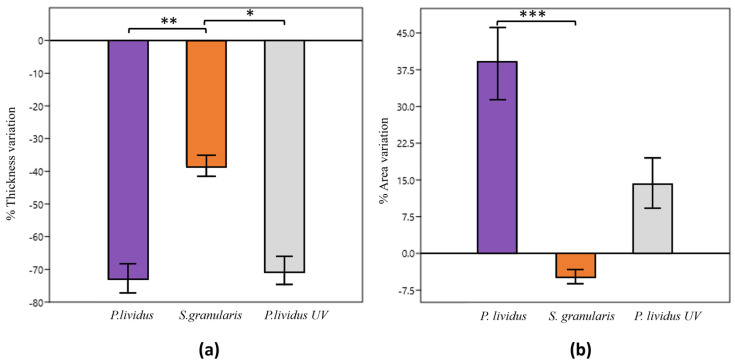
(**a**) Percentage variation of the scaffold thickness after immersion in PBS (means ± st. dev), * *p* < 0.05; ** *p* < 0.005—Kruskal–Wallis + Dunn test. (**b**) Percentage variation of the scaffold area after immersion in PBS (means ± st. dev). *** *p* < 0.001—Kruskal–Wallis + Dunn test.

The percentage of thickness variation of non-crosslinked and crosslinked *P. lividus* scaffolds was significantly higher (Kruskal–Wallis, *p* < 0.05) than that of *S. granularis* scaffolds, which are, therefore, significantly more stable. The thickness variation was negative, indicating a decrease in thickness for all the scaffolds. The percentage area variation of the *S. granularis* scaffolds was significantly lower (Kruskal–Wallis, *p* < 0.001) than that of *P. lividus* scaffolds, again suggesting a greater stability. Crosslinked *P. lividus* scaffolds did not show significantly different values from either of the other two groups considered. *P. lividus* scaffolds (both cross-linked and non-cross-linked) always showed an increased area, whereas *S. granularis* scaffolds showed a decreased area (Figure 9).

#### 2.2.6. Mechanical Tests

**Figure 10:**
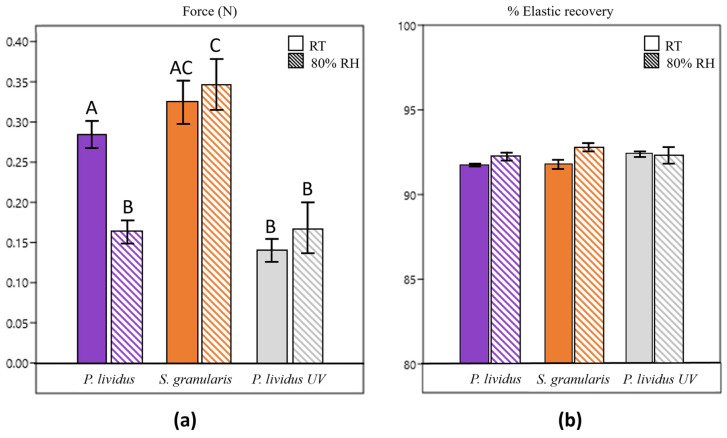
(**a**) Scaffolds’ stiffness (mean ± st. dev) expressed in force (newtons) under different conditions (RT: scaffolds’ stiffness after conditioning at room temperature; 80% RH: scaffolds’ stiffness after conditioning in a climatic chamber at 37 °C and 80% relative humidity). Same letter on the bar (A,B or C) corresponds to no statistically significant difference. Different letters correspond to statistically significant difference (*p* value between A and B < 0.05, *p* value between B and C < 0.05). (**b**) Scaffolds’ elastic recovery (mean % ± st. dev) expressed in % under different conditions (RT: scaffolds’ stiffness after conditioning at room temperature; 80% RH: scaffolds’ stiffness after conditioning in a climatic chamber at 37 °C and 80% relative humidity).

Resistance to compressive stress (stiffness) of the scaffolds ranged from 0.14 N to 0.35 N (Figure 10).

Collagen scaffolds derived from *S. granularis* showed higher resistance than *P. lividus* scaffolds (both non-crosslinked and crosslinked), especially under humid conditions. Non-crosslinked *P. lividus* scaffolds showed significantly (Kruskal–Wallis, *p* < 0.05) different stiffnesses in the two conditions: similar resistance to *S. granularis* scaffolds at RT (room temperature) but more similar resistance to their crosslinked counterparts at 80% RH. The other samples maintained similar resistance regardless of the condition.

Contrary to expectations, cross-linked *P. lividus* scaffolds were significantly less resistant than the others at RT. In humid conditions, they displayed similar stiffness to non-crosslinked *P. lividus* scaffolds but statistically lower resistance (Kruskal–Wallis, *p* < 0.05) than *S. granularis* scaffolds.

Elastic recovery (Figure 10), initially expressed in kPa, ranged from 1.5 kPa to 5.7 kPa. Regardless of the conditions, elastic recovery was higher than 90% in all samples.

## 3. Discussion

### 3.1. PHNQs

Given the biodiversity of edible sea urchin species commercially available, our research aimed to explore the potential of using waste from different species as an environmentally friendly source of antioxidants, collagen, and collagen-based biomaterials. Focusing on the PHNQs extraction yield results (Figure 1), the same protocols optimized in previous works for *P. lividus* similarly allowed the successful extraction of PHNQs from *S. granularis* waste. The yield of *S. granularis*, which was significantly lower (Mann–Whitney, *p* < 0.05) than that of *P. lividus*, suggested that *P. lividus* waste might be a better antioxidant source in terms of extractable amount. The yield from *S. granularis* with white spines was lower than that from purple spines, likely due to the present of less pigmentation in the white variant. Given the PHNQs yields, the total content of PHNQs was in the range of 400–900 mg _PHNQ_/kg _dried test/spines_ in *P. lividus* and in the range of 100–500 mg _PHNQ_/ kg _dried test/spines_ in *S. granularis*. The PHNQ content of the sea urchin species in this study was higher than that reported for species from the Pacific Ocean (11.9 ± 0.7–331.2 ± 5.1 mg/kg) [7] or the Indian Ocean (<60 mg/kg) [8] but lower than that reported for species from the Vietnam Sea (1914 ± 340 – 6151 ± 510 mg/kg) [47], confirming a certain variability in the presence of PHNQ secondary metabolites.

Several factors may influence the production of these antioxidants, including the geographical area where sea urchins are harvested. For example, many studies have shown that different edible species from different parts of the world, such as the Vietnam Sea [47], Madagascar Sea [8], or Okhotsk Sea [7], appear to contain different amounts of PHNQs.

The depth at which sea urchins live may also influence the intraspecific production of PHNQs. For example, in a study conducted by Vasileva et al. (2017) on the edible sea urchin *Strongylocentrotus droebachiensis*, it was found that individuals collected at a depth of 1 meter had significantly lower contents of PHNQs than samples collected at higher depths (85 meters). However, sea urchins collected at the same depths generally showed similar contents of antioxidant molecules [7]. Overall, more research is needed to understand the specific correlation between the habitat of sea urchins and the variability in PHNQ extraction yields from different species.

Focusing instead on the type of PHNQs, both species in this study produce the same PHNQs, except for spinochrome B (exclusive to *P. lividus*) and spinochrome D (exclusive to *S. granularis*), which seem to be species-specific. The numerous extractions of *P. lividus* performed from different batches over the years (Table 1) showed that the ratio of PHNQs can vary significantly. However, our results agree with those of Nhu Hieu et al. (2020) who observed that the most abundant PHNQs in each sea urchin species (e.g., EchA/SpA and spB for *P. lividus* and EchA/SpA for *S. granularis*) are always found regardless of the extraction performed, while the less abundant PHNQs often show greater variations or may be absent [47]. Accordingly, in their study, Anderson et al. (1969) also found EchA, SpA, and SpB in *P. lividus* and EchA/SpA in *S. granularis* [14]. Aiming at finding a correlation between PHNQs relative abundances and further characteristics of each extraction batch, the antioxidant activity was measured for each extract.

The EC_50_ (i.e., the antioxidant activity index) of the *S. granularis* extracts, regardless the spine colour of the sea urchin, is generally an order of magnitude lower (meaning an increased antioxidant activity) than that of *P. lividus*, and it is comparable to that of Trolox^®^ (0.0035 ± 0.0005 mg/mL). Similar EC_50_ values in white and purple *S. granularis* samples are probably due to the presence of the same chemical species of PHNQs (EchA, spA, spD, and spE), although there is a slight variation in their relative ratio. Interestingly, the antioxidant activity appears significantly influenced by the relative amount of SpE regardless of the sea urchin species considered—the higher the percentage of SpE, the greater the antioxidant activity. For example, in the case of *P. lividus*, it is shown that at high percentages of SpE, the EC50 decreases by an order of magnitude (Table 1, *P. l*_28.03.2023), giving a similar result to that of *S. granularis*. However, it was more common for *P. lividus* extracts to have low percentages of SpE and therefore ten times lower antioxidant activities. 

Accordingly, it has been shown in the literature that the antioxidant potential of PHNQs increases with an increasing number of double bonds, hydroxyl, and acetyl groups. Such chemical structures are more likely to be powerful antioxidants donating hydrogen atoms to radical agents. As shown in their study, Brasseur et al. (2017) determined the EC_50_ of isolated spinochromes from the edible sea urchin species *Echinometra mathaei*: the antioxidant activity was similar or superior to Trolox^®^, in the order SpE > EchA/SpC > Trolox^®^ > SpA > SpB (in their work, EchA/spC were co-eluates, and spinochrome D was not included), [8]. Thus, the antioxidant activity was well correlated with the number of hydroxyl groups present in each chemical structure: SpE has six hydroxyl groups compared to the five of EchA. SpA has four hydroxyl groups and one acetyl group, and SpB has only four hydroxyl groups. Similarly, SpD, which has five hydroxyl groups, should be slightly less antioxidant than Spinochrome E.

Another study by Li et al. (2013) showed that a mixture of pigments containing spE, D, and B extracted from the edible sea urchin species *Glyptocidaris crenularis* was more antioxidant than an extract at the same concentration from the edible sea urchin species *Strongylocentrotus intermedius,* which contained only SpB [11]. Therefore, they concluded that spE and spD were expected to have stronger antioxidant activities. Nhu Hieu et al. (2020) found that extracts from the edible sea urchin *Diadema setosum* with high EchA content were significantly more antioxidant than those from other sea urchin species without EchA [47]. According to what has been said so far, *P. lividus* mixtures often have lower percentages of SpE and therefore lower antioxidant activities. In *S. granularis*, however, the constant presence of high percentages of spE, spD, and EchA/spA (usually higher than those found in *P. lividus*) is responsible for such high antioxidant activity. Overall, despite a lower extraction yield, this promising antioxidant activity makes PHNQs from *S. granularis* as interesting and potentially useful for future biomedical applications as those from *P. lividus*.

### 3.2. Collagen

Previous research demonstrated that collagen can be extracted in its truly native structure (fibrils still decorated with proteoglycans/glycosamminoglycans (e.g., chondroitin sulphate) from several sea urchin mutable collagenous tissues, including the peristomial membrane. Characterization of this collagen by different methods, such as SDS-PAGE, FT-IR and Raman microscopy, suggest a similarity with mammalian collagen type I [22,48], which is the main collagen type in human tissues, such as—for example—the dermis. This further supports the utility of sea urchin species as a potential source of collagen for biomedical applications and encourages the research to maximize the “green” extraction of this valuable molecule from the waste of different sea urchin species in order to maximise the potential use of the total waste produced worldwide.

In line with this approach, the results here obtained showed that the same protocol used for *P. lividus* can be successfully applied to other edible species, such as *S. granularis*. However, the collagen extraction yield of *S. granularis* (expressed as the percentage of collagen extracted from a single membrane) was lower than that of *P. lividus* but still in line with those reported in the literature for other marine organisms. For example, Huang et al. (2011) obtained collagen yields of 4% and 19% (relative to fresh weight) from the skin of the pufferfish *Diodon holocanthus* using acid or pepsin solutions, respectively [34]. Similarly, Singh et al. (2011) extracted collagen from the skin of pangasius *Pangasianodon hypophthalmus* using acid and pepsin solutions with extraction yields of 5.1% and 7.7% (both relative to fresh weight), respectively [35]. In this context, it must be underlined that our extraction method is specifically designed to preserve the full integrity of collagen fibril, this possibly reducing the extraction yields if compared to acid or enzymatic extractions, which lead to a non-native fibrillar collagen.

It should be noted that the peristomial membranes of the new species provided by the restaurants were sufficient for only one extraction, whereas the collagen yield of *P. lividus* is an average of several extractions. Therefore, the data obtained should be considered with caution. Given the large size/thickness of the peristomial membranes of *S. granularis*, it is possible that the extraction efficiency could have been reduced. Longer disaggregation times or higher concentrations of extraction solution might improve the collagen yield of *S. granularis*. This observation suggests the importance of species-specific protocol optimisation in maximising collagen extraction. 

Once the possibility of using *S. granularis* waste as a source of collagen was confirmed, it was necessary to investigate the possibility of using it to produce optimal biomaterials for biomedical applications (especially regenerative medicine), similar to what was done with *P. lividus* collagen [23,24]. According to the images obtained by scanning electron microscopy (SEM), the collagen of both sea urchin species produces scaffolds with interconnected pores of appropriate size, creating a three-dimensional network that mimics the extracellular matrix, thereby promoting cell infiltration, vascularisation, and the flow of nutrients and oxygen to allow tissue regeneration [49]. However, *S. granularis* scaffolds showed more regular and denser pores in cross section, whereas *P. lividus* showed a less compact structure.

Since fibril diameter and scaffold porosity are factors that likely influence the stability and mechanical performance of the biomaterial, these two characteristics were assessed. The fibril diameter (109 to 441 nm) of *S. granularis* collagen is consistent with that of *P. lividus* (64 to 442 nm), which is in line with the typical fibril sizes of marine collagen (25–300 nm) reported in the literature [22,23] and similar to mammalian fibres [50]. However, it is noteworthy that the mean diameter was significantly higher in *S. granularis,* which may potentially contribute to the slightly different scaffold internal structure compared to that of *P. lividus*.

Porosity was estimated as the percentage of macroporosity, which provides a rough indication of the volume of the biomaterial potentially colonisable by cells. A porosity greater than 90% is considered optimal for cellular infiltration, proliferation, neovascularisation, and oxygen and nutrients diffusion required during tissue formation [51].

Despite the slight differences observed in the internal organisation, the macroporosity was comparable between the two species and was consistent with what is known in the literature for collagen scaffolds [52,53]. 

The scaffolds were further characterised in terms of degradation, hydration and mechanical tests to assess their suitability for applications in tissue regeneration. In addition, ensuring that each scaffold has consistent and reproducible properties is useful for future industrial production of these biomaterials.

The degradation test was designed to replicate physiological conditions in vivo, as these biomaterials naturally biodegrade as new tissue forms in wounds. Thus, a saline solution (PBS) and collagenase were used to simulate different physiological conditions of tissue healing. In particular, collagenase was chosen at a concentration of 0.01 mg/ml, as reported in the literature for this type of test [54]. It is known that the biodegradation of a biomaterial must be optimal for the type of tissue/application for which it is used and must be appropriately calibrated to coincide with the formation of neo-tissue [55,56]. *S. granularis* scaffolds degraded more slowly in PBS than *P. lividus* scaffolds, similar to the degradation kinetics of cross-linked *P. lividus* scaffolds. The latter were used as a positive control to compare the naturally stable *S. granularis* scaffolds with those of the other species artificially stabilised by a physical process. Indeed, UV radiation is known for its cross-linking capacity [57,58]. As scaffolds with a higher number of pores are known to degrade more slowly [59], the higher stability of *S. granularis* scaffolds (similar to the cross-linked ones) is likely due to the denser pores observed by SEM.

As for degradation in enzymatic condition, both types of scaffolds showed very similar kinetics and were significantly different from crosslinked scaffolds. This difference can be attributed to the crosslinking process, which increases the stability to enzymatic degradation of crosslinked scaffolds compared to scaffolds made of collagen alone [57,58,60].

The hydration test was used to determine the water uptake of the scaffold and to assess any dimensional changes in an aqueous environment, mimicking a wound.

High water uptake is a positive feature as it allows for a more faithful mimicry of the extracellular matrix, promoting cell migration, growth, organisation, and angiogenesis during tissue regeneration and wound healing [49,61]. Water uptake can depend on both the porosity of the scaffold and the chemical nature of the collagen—as previously discussed, high scaffold porosity results in a greater capacity to retain water.

Regarding the chemical nature of the material, sea urchin collagen is highly hydrophilic because it is extracted in a way that naturally preserves its glycosaminoglycan (GAG) content. GAGs have the ability to retain water, facilitating its absorption [62]. This property is likely the reason why the water uptake of the tested scaffolds is much higher compared to similar collagen scaffolds in the literature [63,64]. On the other hand, other studies show water uptake for collagen–gelatine scaffolds in line with our results [61]. The origin of the collagen may significantly influence the results. As the volume absorbed by the macropores is similar for all scaffolds in our study, the water uptake did not show a significant difference.

In tissue engineering, it is useful to know the dimensional change in aqueous environment to design specific scaffolds for in vivo use, as it provides an idea of their dimensional changes when in contact with blood and fluids. In the present work, the produced scaffolds consistently showed a decrease in thickness, indicating a general thinning of the collagen structure, regardless of the sea urchin species. However, in case of *S. granularis*, the reduction in thickness was significantly less (−38%). These data suggest greater structural stability than *P. lividus* scaffolds (average thickness variation of –73%) and UV-cross-linked *P. lividus* scaffolds (average thickness variation of –71%). It is worth noting that crosslinking does not appear to stabilise the overall thickness of *P. lividus* scaffolds particularly well, probably because the crosslinking method is more effective at stabilising area variation than thickness. This problem (dimensional reduction) has indeed been encountered and successfully addressed in recent in *vivo* rat skin regeneration tests using these innovative sea-urchin-derived biomaterials [25].

The percentage area variation was significantly different between *S. granularis* and *P. lividus* scaffolds. Even though the area has increased for *P. lividus* scaffolds and reduced for *S. granularis* scaffolds, the latter result more stable. It is hypothesised that the larger average fibril diameter of *S. granularis* and the presence of denser pores contribute to less alteration of the three-dimensional structure (observable in both thickness and area variations) and thus to greater stability. 

Finally, mechanical tests were carried out to further characterise the collagen scaffolds by investigating their ability to bear compressive loads that can be naturally present in biological environments. All the scaffolds were tested at both room temperature and 80% humidity (at 37 °C), replicating the situation that most closely resembles a humid environment, such as a wound.

Our stiffness results ranged from 0.14 N to 0.34 N. They are comparable to collagen-rich tissues such as human dermis, rat tendons, and murine liver, whose estimated stiffness ranges from 0.06 to 0.86 N/mm^2^ [65]. In general, providing a more detailed comparison with human skin is challenging because the literature reports a wide range of skin stiffness values spanning three orders of magnitude (from 1 N/mm^2^ to 1000 N/mm^2^) depending on the different methods used, the anatomical region tested, the donor’s age, and the hydration level of the skin [65,66,67,68].

Non-crosslinked *P. lividus* scaffolds showed a significant decrease in stiffness at humid conditions. It is known that humidity can affect the collagen structure, making it less resistant [69]. However, *S. granularis* scaffolds were equally resistant at RT and 80% RH. A higher resistance was also observed in humid conditions compared to all other samples. Such behaviour can be attributed to the denser internal pore organisation, which is likely to provide greater structural stability to the whole scaffold, maintaining stiffness and mechanical properties [70], also in humid condition.

Crosslinked *P. lividus* scaffolds showed lower resistance than other groups, especially at room temperature. This finding contrasts with the typical effect of UV radiation, which is known to improve scaffold stability and mechanical properties. Although mechanical properties were not improved in this study, UV light still confers stability, as indicated by the similar resistance of the crosslinked scaffolds in both scenarios tested.

Given the importance of the internal structure of the scaffolds, it is likely that many other factors not considered in this study, such as the homogeneity or heterogeneity of the pores, their morphology and the orientation of the fibrils, will need to be considered in the future [71].

Finally, all the scaffolds tested, regardless of the fabrication conditions, showed an elastic recovery of more than 90% and no significant difference was observed, indicating their ability to withstand the applied stress without experiencing permanent deformation. Our results seem promising, as the results were expressed in kPa, ranging from 1.5 to 5.7 kPa, which are comparable to the average values of human skin elasticity reported in the work of Yang et al. (2018): from 3.5 to 15.5 kPa for abdominal skin, from 3.5 to 26.0 kPa for forearm, from 6.6 to 28.9 kPa for chest wall, and from 12.1 to 48.4 kPa for finger [72]. Other mechanical properties will be investigated in the future.

Overall, *S. granularis* collagen appears to be as equally promising for scaffold production as *P. lividus* collagen depending on the intended applications. These differences could indeed contribute to a future diversification of applications. For example, collagen that allows the production of more stable scaffolds would be better suited to regenerative medicine contexts where slow biomaterial degradation is required, such as bone regeneration [73] or severe wound healing. On the other hand, collagen which allows the production of less stable scaffolds, may be better suitable for skin wounds and burns, where tissue regeneration is faster [74], or even in cosmetics or pharmaceuticals fields (e.g., cream production).

## 4. Materials and Methods

### 4.1. Chemicals

Formic acid, ethyl acetate, ethanol, methanol, UPLC-grade acetonitrile were from Carlo Erba Reagents (Italy). Anhydrous sodium sulphate, 2,2’-azino-bis(3-ethylbenzothiazoline-6-sulfonic acid), ammonium persulfate, Tris-HCl, EDTA, phosphate-buffered saline, SDS, β-mercaptoethanol, NaCl were from Sigma Aldrich.

### 4.2. PHNQs Extraction 

A mixture of PHNQs was extracted using a protocol previously optimised by Marzorati et al. (2021) [5]. Briefly, PHNQs were extracted from lyophilised and powdered waste of both sea urchin species previously collected from restaurants close to the University of Milan and stored at −20 °C. For each gram of material, 3.5 mL of 6M formic acid solution was added dropwise to a beaker. The reaction was kept under stirring conditions for 2 hours. The resulting solution was centrifuged at 6000 rpm for 5 minutes and the supernatant was further filtered using a Buchner funnel. To isolate the PHNQs from undesirable co-extracted compounds, liquid-liquid extraction was performed three times with ethyl acetate (150 mL). The orange-pink organic phase was repeatedly washed with distilled water (150 mL) to remove inorganic salts. After about 10 washing cycles, anhydrous sodium sulphate was added to completely remove water. The resulting solution was filtered through cotton and dried using a rotary evaporator (Buchi, Italia srl) and a mechanical vacuum pump.

The following formula was used to calculate the PHNQs extraction yield:

(g_extracted PHNQs_/g_test powder_) × 100 (1)

Five extractions were carried out for *P. lividus* and, due to lack of material, three extractions were carried out for *S. granularis* with purple spines and two were carried out for *S. granularis* with white spines.

### 4.3. Ultra-High-Pressure Liquid Chromatography–Mass Spectrometry

A UPLC-PDA-ESI-HR-MS equipment was used to identify the PHNQs in the extracts by determining their molecular weights (*m*/*z*). LC instrument: ACQUITY UPLC I-Class (Waters, Milford, MA, USA). Column: ACQUITY UPLC BEH C18 (100 × 2.1 mm, 1.7 µm) fitted with a VanGuard cartridge (Waters, Milford, MA, USA). Column temperature: 34 °C. Eluents: A, water + 0.1% formic acid; B, acetonitrile + 0.1% formic acid. Flow rate: 0.4 mL/min; gradient: START: 95% A: 5% B; t = 12.90 min: 60% A: 40% B; t = 15.00 min: 10% A: 90% B; t = 15.50 min: 10% A: 90% B; t = 16.00 min: 95% A: 5% B; t = 20.00 min: 95% A: 5% B. Injection volume: 2-10 μL depending on sample concentration to optimise signal. PDA detector: ACQUITY UPLC PDA Detector (Waters, Milford, MA, USA). Wavelength range: 210–500 nm. Mass spectrometer: Synapt G2-Si QToF (Waters, Milford, MA, USA). High-resolution mode. Ionisation source: ZsprayTM ESI-probe (Waters, Milford, MA, USA). Ionisation polarity: negative. Acquisition mass range: 50–600 *m*/*z*. MS/MS fragmentation mode: CID. Lock mass compound: leucine enkephalin. Data elaboration software: MassLynx v4.2 (Waters, Milford, MA, USA). 

The relative ratios of the PHNQs were determined by comparing the areas under the peaks of the chromatograms at 445 nm obtained using the following method: instrument: Acquity UPLC equipment (Waters, Milford, MA, USA). Column BEH C18, 2.1 × 50 mm, 1.7 μm. Column temperature: 34 °C. Eluent A: water + 0.1% formic acid; eluent B: acetonitrile + 0.1% formic acid. Flow rate: 0.2 mL/min; gradient: START: 95% A: 5% B; t = 25 min: 10% A: 90% B; t = 27 min: 95% A: 5% B; t = 30 min: 95% A: 5% B. Injection volume: 2 μL. Sample temperature: 20 °C. Each sample was injected three times. Wavelengths monitored for PHNQs absorption: 445 nm. 

### 4.4. ABTS Assay

The ABTS^•+^ assay (2,2’-azino-bis(3-ethylbenzothiazoline-6-sulfonic acid)) was performed to evaluate the antioxidant activity. Trolox^®^ (EC50: 0.0035 ± 0.0005 mg/mL) was used as a reference as it is a vitamin E analogue well known in the literature as a potent antioxidant. A methanolic solution of PHNQs extract (1 mg/mL) and a water solution of ABTS (by weighing 3 mg of ammonium persulfate and 19 mg of powdered ABTS in 5 mL of distilled water) were prepared and kept in the dark overnight.

The following day, the ABTS^•+^ solution was diluted in ethanol to reach an absorbance of 0.7 AU at 734 nm (about 1:75 *v*/*v*). Finally, cuvettes containing 1.2 mL of the diluted ABTS^•+^ solution and increasing concentrations (in the range 0.02–0.1 mg/m) of the PHNQs samples were prepared. The cuvettes were kept in the dark for 1 hour, and the absorbance of each was then read at 734 nm using a spectrophotometer. The blank did not contain the sample.

The obtained values of % ABTS^•+^ remaining, calculated as:

% ABTS^•+^ remaining = (A_734 nm, 1h, sample_/A_734 nm, 1h, blank_) % (2)

These values were graphically plotted vs. the concentration of the antioxidants. PHNQs extract EC50 values were read on the graph.

### 4.5. Collagen Extraction

Collagen was extracted using a protocol previously developed and optimised in our laboratory using *P. lividus* as model species [22,23,24]. Briefly, in the present study, the peristomial membranes of both species were isolated from the waste, rinsed in seawater, weighed, and divided into Falcon tubes. A hypotonic buffer (Tris-HCl 10mM, EDTA 0.1%; pH 8 in distilled water; 1 mL per 0,01 g of membranes) was added, and the falcon tubes were placed on a rotary shaker disc at 23 °C overnight. The following day, three brief washes were performed in PBS (phosphate-buffered saline; pH 7.8) and a decellularizing solution (Tris-HCl 10 mM, SDS 0.1%; pH 8 in distilled water) was added (1 mL per 0.01 g of membranes). The samples were left on a rotary shaker disc overnight at 23 °C. Several washes in PBS were performed to remove the sodium dodecyl sulphate, and a disaggregating solution (0.5 M NaCl, 0.1 M Tris-HCl pH 8.0, 0.1 M β-mercaptoethanol, 0.05 M EDTA) was added and left on a rotary shaker disc at room temperature for 5 days. After these procedures, which caused almost complete disaggregation of tissue, the obtained collagen suspension was filtered through a steel mesh filter (0.2 mm) and dialysed against 500 mL of 0.5 M EDTA solution (pH 8) for 4 hours and against distilled water overnight. Finally, the suspension in distilled water was stored at −80 °C until use.

Collagen concentration (mg/mL) was measured by drying and weighting a known aliquot of collagen suspension. The total collagen (g) was determined as collagen concentration (mg/mL) × total extracted volume (mL). Finally, the collagen extraction yield was measured for each species as follows:

[total collagen (g)/total peristomial membranes fresh weight (g)] × 100(3)

Several extractions were performed from *P. lividus*, whereas only one extraction could be performed from the peristomial membranes of *S. granularis* due to difficulties in sourcing from restaurants where *P. lividus* is the more commonly used species.

### 4.6. Production of 3D Scaffolds

Collagen suspension was centrifuged at 1700 g for 5 min and resuspended in 6% (*v*/*v*) EtOH/water solution to obtain a 6 mg/mL suspension. The 3D scaffolds were produced by adding 1 mL of this collagen suspension into rubber silicone molds (diameter 1 cm, height 1 cm). The samples were frozen at −80 °C overnight and freeze dried (Edwards Pirani 1001) overnight.

### 4.7. Ultrastructural Analysis: Scanning Electron Microscopy (SEM)

The scanning electron microscope (FE-SEM Sigma microscope ZEISS, Germany) was used to investigate the ultrastructural features of 3 scaffolds per species. Samples were mounted on stubs and gold coated (Nanothec Semprep 2). The upper surface, longitudinal section, and cross section of each scaffold was observed, and the diameters of 100 collagen fibrils were measured using ImageJ.

### 4.8. Macroporosity

The macroporosity was calculated by the water squeezing method [75]. Briefly, 3 scaffolds per species were individually equilibrated in PBS for one hour and weighed (P1) before being squeezed to remove the water filling the pores and weighed again (P2). The macroporosity volume was calculated using the following:

Macroporosity (%) = (P1 − P2) ×100/P1 (4)

### 4.9. Degradation Tests

Collagen scaffolds (4 per sea urchin species) were weighed, immersed in 4 mL of PBS, and incubated at 37 °C. The remaining mass (% remaining mass) was calculated as:

% remaining mass = (initial weight − final weight) × 100/initial weight (5)

The remaining mass was measured at two different time points: 1 day and 10 days. At each time point, the scaffolds were repeatedly washed in distilled water to remove residual salts, frozen at −80 °C, lyophilized, and then weighed again.

The same procedure was repeated for scaffolds immersed in collagenase solution (0.01 mg/mL). The remaining mass were measured at two different time points: 6 h and 24 h.

In both tests (PBS and collagenase), 4 crosslinked *P. lividus* scaffolds artificially crosslinked by physical method (UV) were used as an additional control to compare *S. granularis* scaffolds with those of the other species artificially stabilised.

### 4.10. Hydrations Tests

Five scaffolds per sea urchin species were photographed a mobile phone, weighed, immersed in 4 mL of PBS, and incubated at 37 °C for 3 hours. They were then photographed and weighed again. Area and thickness measurements were performed using the ImageJ software (1.54 h version). Water uptake, percent thickness variation, and percent area variation were calculated using the following formulas:

Water uptake = (final weight − initial weight) × 100/initial weight(6)

% thickness variation = (final thickness − initial thickness) × 100/initial thickness(7)

% area variation = (final area − initial area) × 100/initial area(8)

Five UV-crosslinked *P. lividus* scaffolds were used as an additional control to compare *S. granularis* scaffolds with those of the other species artificially stabilised by a physical process.

### 4.11. Mechanical Tests

Two consecutive compression cycles were performed using a dynamometer (mod. Z005, Zwick Roell, Ulm, Germany) fitted with a 100 N load cell and connected to two plates (base: 150 mm diameter, compression element: 30 mm diameter) placed at a distance of 22 mm apart. Each compression cycle accounted for a maximum deformation of the sample of 2 mm, at a speed of 2 mm s^−1^. Scaffolds (5 per species) underwent compressive test after conditioning at room temperature or storage in a climatic chamber (37 °C and 80% relative humidity, RH).

In both test scenarios, 5 UV-crosslinked *P. lividus* scaffolds were used as an additional control to compare *S. granularis* scaffolds to those of the other species artificially stabilised by a physical process.

Finally, a force–time profile was recorded, and the following parameters were elaborated by TestXpert V10.11 Master software: resistance to compressive stress (expressed as maximum compressive force in N) and elastic recovery (expressed as kPa). 

### 4.12. Statistical Analyses

For two small sample sizes, the Mann–Whitney non-parametric statistical test was used to analyse the PHNQs extraction yields, the diameter of collagen fibrils, the percentage of macroporosity, and the mechanical test results.

For three small sample sizes, the non-parametric Kruskal–Wallis test and Dunn’s multiple comparison test were used to analyse the degradation, hydration, and mechanical test results.

## 5. Conclusions

Considering the biodiversity of edible sea urchins that are commercialised every year, this work aimed to investigate whether the wastes of different species could be eco-friendly sources of antioxidants, collagen, and collagen-based biomaterials as already demonstrated for *P. lividus* [4,5,22,23,24]. 

The same extraction protocols for collagen and PHNQs were applied to *S. granularis* waste, resulting in lower yields than *P. lividus*. The protocols need to be improved and optimised in the future and adapted to different sea urchin species. 

Despite variable extraction yields, partially species-specific mixtures of PHNQs were obtained. To specifically understand the origin of the variability in the PHNQ yields and relative ratio, further investigations would be necessary to correlate extraction data with different collection areas, depths, or seasonal periods. The PHNQ antioxidant activity was particularly dependent on the presence of Spinochrome E. Spinochrome E was frequently and consistently found in *S. granularis*, making it an optimal species for obtaining PHNQ mixtures with high antioxidant activity.

The produced collagen-based scaffolds of the two sea urchin species showed similar ultrastructural features. *S. granularis* scaffolds had denser pores and larger collagen fibrils, resulting in higher stability. Overall, this study highlights the potential to successfully exploit waste from different sea urchin species for the sustainable production of valuable bioactive or structural compounds and biomaterials. The observed differences in PHNQs antioxidant activity or scaffolds properties may lead to different applications and diversification of use in biomedical fields.

Open questions relate to the economic feasibility and environmental sustainability of the proposed extraction processes. In order to address this point and build a realistic biorefinery value chain, further studies are ongoing.

## Figures and Tables

**Table 1:** Relative abundances of PHNQs derived from different extracts. The different extractions are dated (labels of entry 1) and come from different batches (*P. lividus, P. l*) or from the same batch (*S. granularis, S. g*, P: purple; W: white). EC_50_ (mg/mL, last entry) in relation to the relative ratios of the PHNQs.

Extraction	% (EchA + SpA)	% SpB	% SpD	% SpE	% Echinamine	EC_50_ (mg/mL)
*P. l*_ 28.03.2023	50	11	0	37	1.5	0.0037
*P. l*_ 11.01.2023	23.6	73	0	1.2	2.8	0.03
*P. l*_ 06.07.2022	26	70	0	1.7	0.2	0.033
*P. l*_12.04.2022	22	72	0	3.9	1.2	0.019
*P. l*_ 10.01.2022	92.5	7.5	0	0	0	0.027
*S. g*_ 08.06.2023_P	90.7	0	4.1	4.6	0.6	0.0034
*S. g*_ 08.06.2023_W	86	0	2.8	11.2	0	0.0041
*S. g*_ 17.05.2023_P	86.4	0	5.1	8	0.5	0.0053
*S. g*_ 17.05.2023_W	75.8	0	1.9	21.8	0.5	0.0068
*S. g*_ 13.02.2023_P	66.4	0	18.9	14.7	0	0.0071

## Data Availability

All data are available upon request.

## Supplementary Materials

The following supporting information can be downloaded at: https://www.mdpi.com/article/10.3390/md22040163/s1, Table S1: Extraction PHNQs yields; Table S2: Relative ratios of PHNQs in each extraction from the two sea urchin species; Table S3: Comparative table of collagen extraction from the two sea urchin species; Table S4: Kruskal–Wallis + Dunn test results of degradation kinetics in PBS at 1 day and 10 days; Table S5: Kruskal–Wallis + Dunn test results of degradation kinetics in collagenase at 6 h; Table S6: Results of Kruskal–Wallis + Dunn and Mann–Whitney tests used to compare stiffness under different conditions; Figure S1: ESI-HRMS spectrum of spE; Figure S2: ESI-HRMS spectrum of spB; Figure S3: ESI-HRMS spectrum of spA; Figure S4: ESI-HRMS spectrum of EchA; Figure S5: ESI-HRMS spectrum of spD; Figure S6: ESI-HRMS spectrum of echinamine.
