# Supplementary material for: Sea Urchin Food Waste into Bioactives: Collagen and Polyhydroxynaphtoquinones from P. lividus and S. granularis
Source: Mar Drugs. 2024 Apr 3;22(4):163. doi: 10.3390/md22040163 (PMC11051063; doi:10.3390/md22040163)

SUPPLEMENTARY MATERIALS

Table S1. Extraction PHNQs yields. *P. lividus* (*P. l*) and *S. granularis* (*S. g*, P: purple; W: white). The different extractions are numbered and were performed from different batches (*P. lividus*) or from the same batch (*S. granularis*).

Extraction	Extraction yield	Mean% ± st. dev
*P. l*_ 28.03.2023	0.08%	
*P. l*_ 11.01.2023	0.08%	
*P. l*_ 06.07.2022	0.04%	0.07 ± 0.02
*P. l*_12.04.2022	0.09%	
*P. l*_ 10.01.2022	0.06%	
*S. g*_ 08.06.2023_P	0.02%	
*S. g*_ 17.05.2023_P	0.03%	0.033 ± 0.02
*S. g*_ 13.02.2023_P	0.05%	
*S. g*_ 08.06.2023_W	0.01%	0.015 ± 0.01
*S. g*_ 17.05.2023_W	0.02%	

Table S2. Relative ratios of PHNQs in each extraction from the two sea urchin species: *P. lividus* (*P. l*) and *S. granularis* (*S. g*, P: purple; W: white). The different extractions are numbered and were performed from different batches (*P. lividus*) or from the same batch (*S. granularis*)

Extraction	EchA + SpA	SpB	SpD	SpE	Echinamine
*P. l*_ 28.03.2023	50	11	0	37	1.5
*P. l*_ 11.01.2023	23.6	73	0	1.2	2.8
*P. l*_ 06.07.2022	26	70	0	1.7	0.2
*P. l*_12.04.2022	22	72	0	3.9	1.2
*P. l*_ 10.01.2022	92.5	7.5	0	0	0
*S. g*_ 08.06.2023_P	90.7	0	4.1	4.6	0.6
*S. g*_ 08.06.2023_W	86	0	2.8	11.2	0
*S. g*_ 17.05.2023_P	86.4	0	5.1	8	0.5
*S. g*_ 17.05.2023_W	75.8	0	1.9	21.8	0.5
*S. g*_ 13.02.2023_P	66.4	0	18.9	14.7	0

Table S3. Comparative table of collagen extraction from the two sea urchin species: *P. lividus* (left) and *S. granularis* (right) weight (g) and diameter (cm) of the peristomial membranes, the collagen extraction yield (%) and the amount of collagen obtained from a single membrane (mg).

**Sea urchin species**	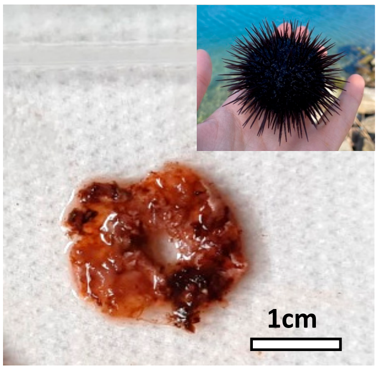	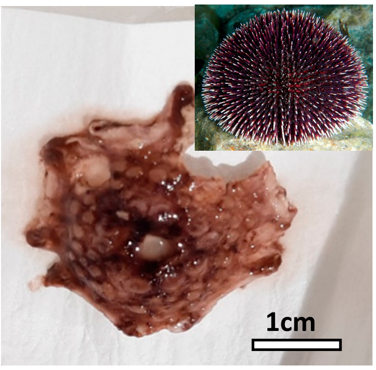
Average single membrane weight (g)	0.09 g	0.4 g
Membrane diameter (cm)	2 cm	3 cm
Extraction yield (%)	10.34 ± 2.5 %	6 %
Collagen from 1 membrane (mg)	7.75 ± 1.3 mg	17 mg

Table S4. Kruskal-Wallis + Dunn test results of degradation kinetics in PBS at 1 day and 10 days. Values are significant at $p < 0.05$.

**Time point**	**Comparison - PBS**	**p value**
1 day	*P. lividus/ S. granularis*	0.002
1 day	*P. lividus/ P. lividus* UV	0.096
1 day	*S. granularis/ P. lividus* UV	0.169
10 day	*P. lividus/ S. granularis*	0.029
10 day	*P. lividus/ P. lividus UV*	0.010
10 day	*S. granularis/ P. lividus UV*	0.692

Table S5. Kruskal-Wallis + Dunn test results of degradation kinetics in collagenase at 6 h. Values are significant at $p < 0.05$.

Time point	Comparison - collagenase	p value
6 h	*P. lividus*/ *S. granularis*	0.433
6 h	*P. lividus*/ *P. lividus* UV	0.006
6 h	*S. granularis*/ *P. lividus* UV	0.049

Table S6. Results of Kruskal-Wallis + Dunn and Mann-Whitney tests used to compare stiffness under different conditions. Only significant values with $p < 0.05$ are shown. RT: room temperature, RH: relative humidity

Statistic test	Conditions	Comparison – compressive stress	p value
Mann - Whitney	RT/ 80% RH	*P. lividus*/ *P. lividus*	0.012
Kruskal Wallis + Dunn test	RT/ RT	*P. lividus*/ *P. lividus* UV	0.028
Kruskal Wallis + Dunn test	RT/ RT	*S. granularis*/ *P. lividus* UV	0.002
Kruskal Wallis + Dunn test	80% RH/ 80% RH	*P. lividus*/ *S. granularis*	0.039
Kruskal Wallis + Dunn test	80% RH/ 80% RH	*S. granularis*/ *P. lividus* UV	0.015

Figure S1 - ESI-HRMS spectrum of spE (m/z=252.9988, [M-H]$^{-}$)

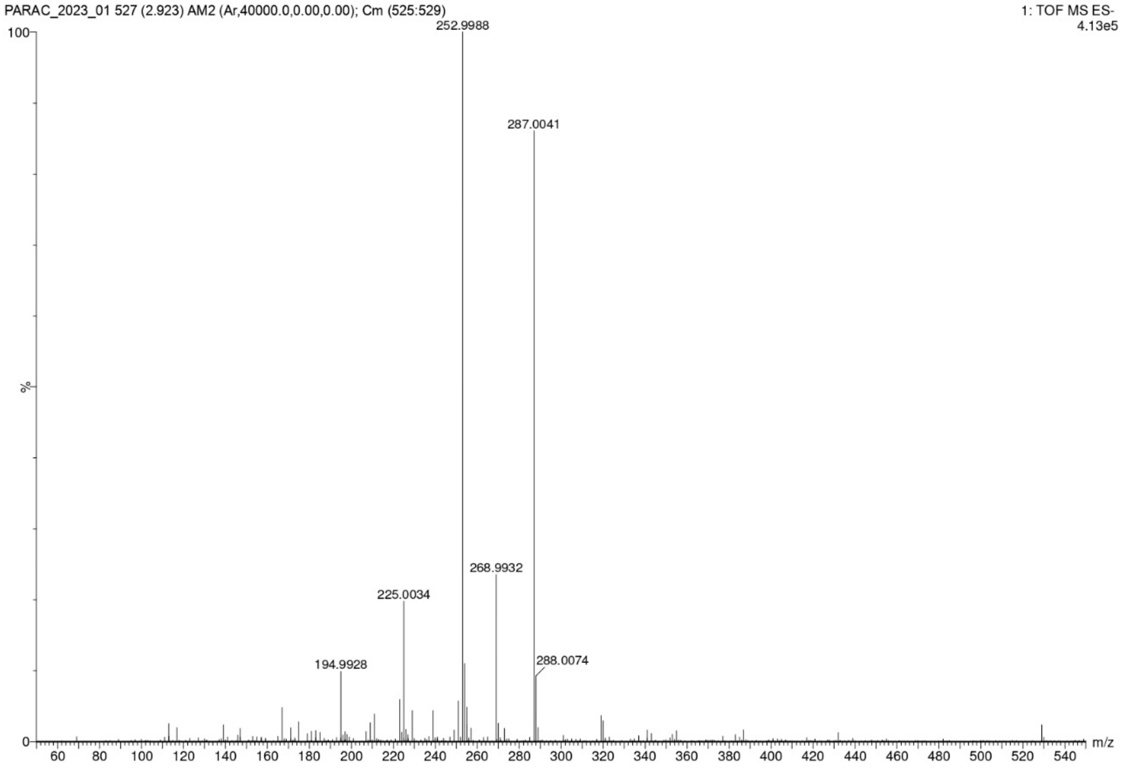

Figure S2 - ESI-HRMS spectrum of spB (m/z=221.0085, [M-H]$^{-}$)

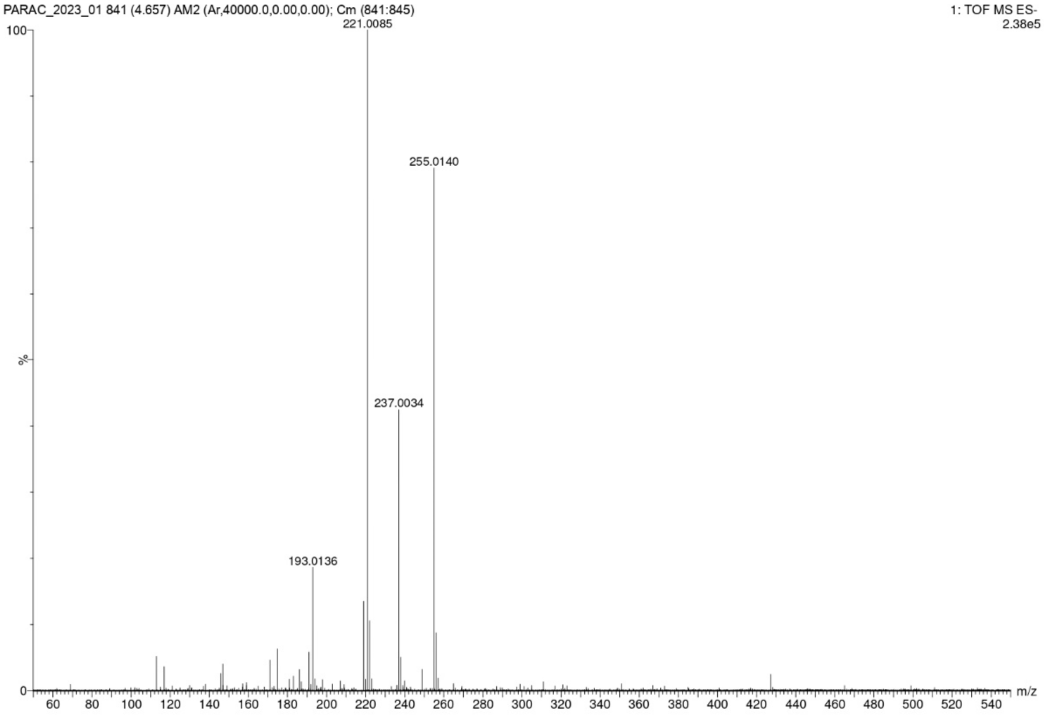

Figure S3 - ESI-HRMS spectrum of spA (m/z=263.0190, $[M-H]^-$)

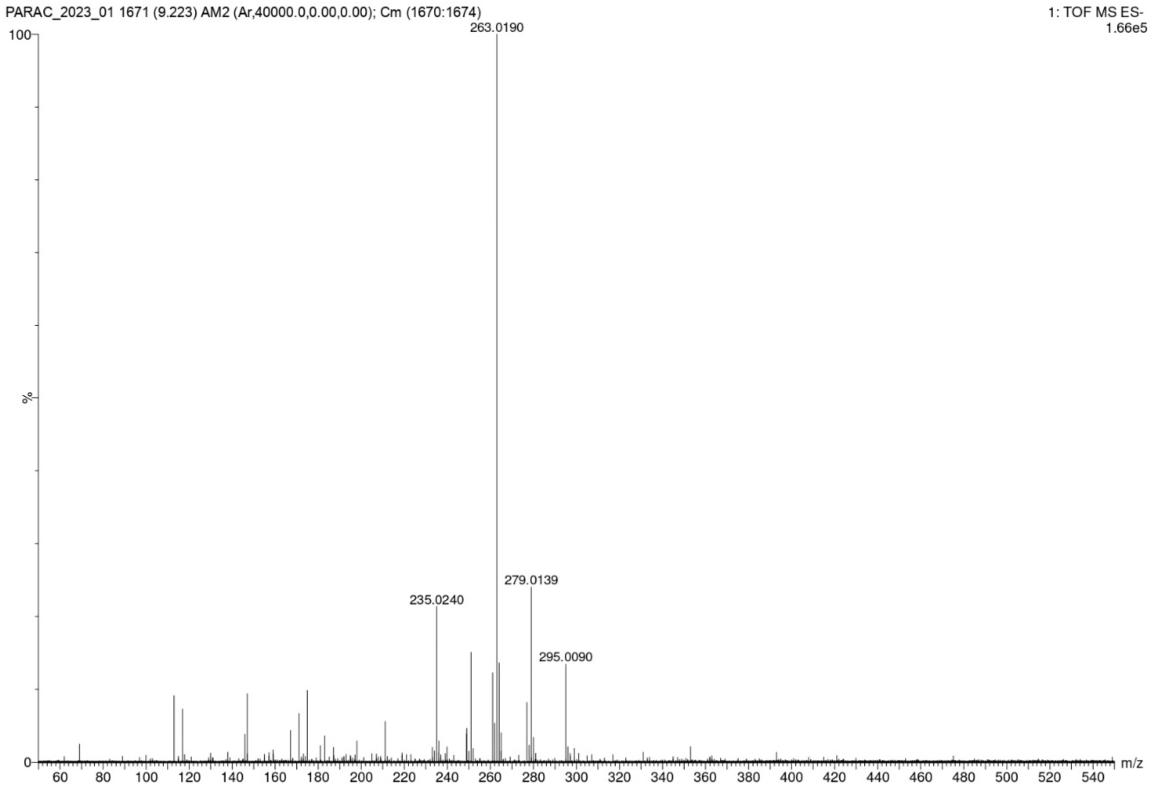

Figure S4 - ESI-HRMS spectrum of EchA (m/z=265.0350, $[M-H]^-$)

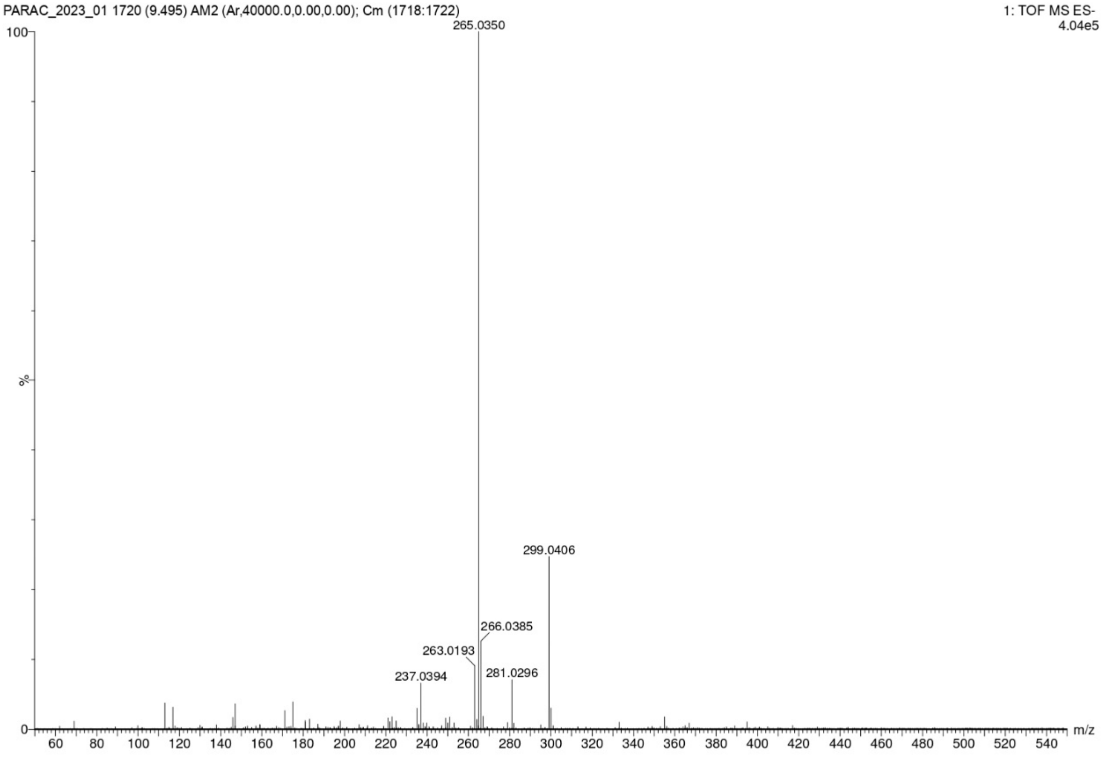

Figure S5 - ESI-HRMS spectrum of spD (m/z=237.0032, [M-H]$^-$)

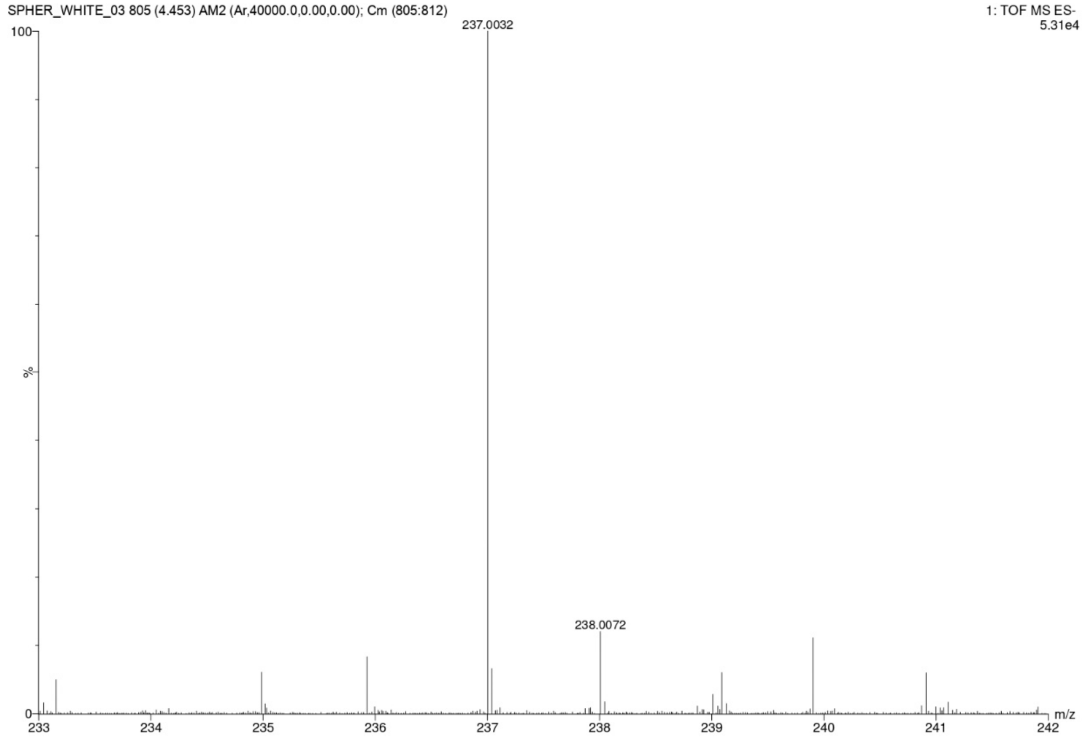

Figure S6 - ESI-HRMS spectrum of echinamine (m/z=252.0145, [M-H]$^-$)

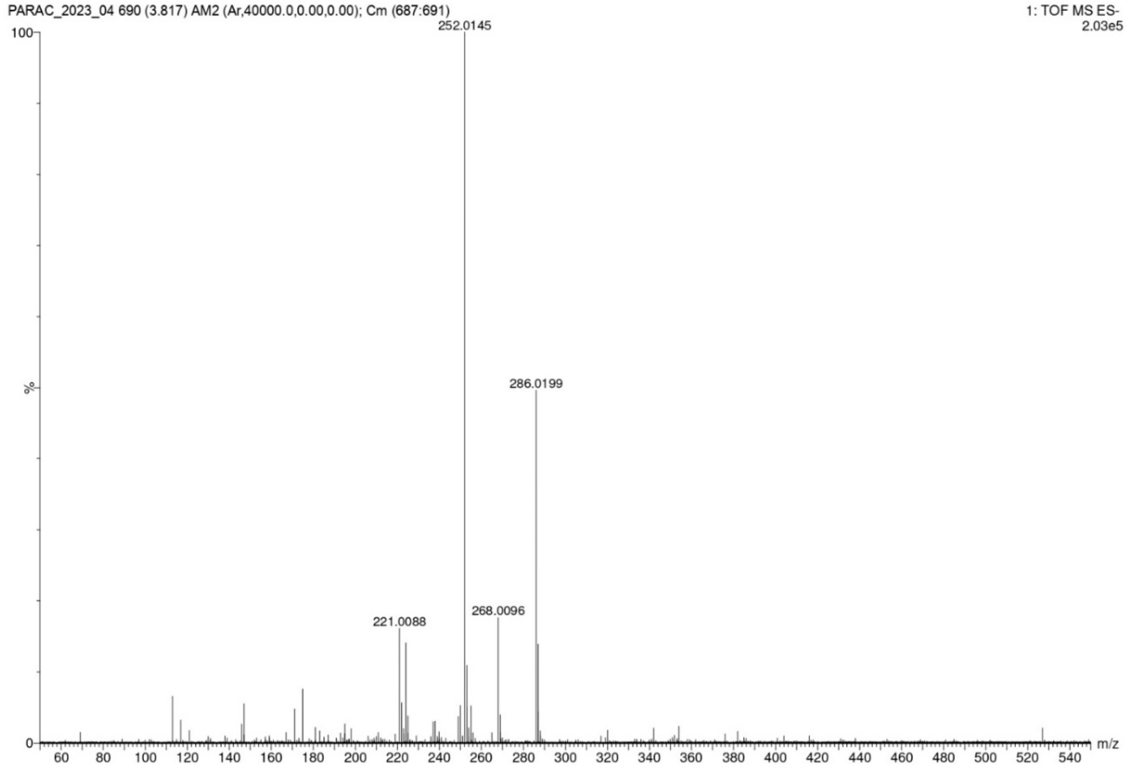

Supplement: Supplementary file 1 [file marinedrugs-22-00163-s001.zip › marinedrugs-2924907-supplementary.pdf]
